# A High Amylose Wheat Diet Improves Gastrointestinal Health Parameters and Gut Microbiota in Male and Female Mice

**DOI:** 10.3390/foods10020220

**Published:** 2021-01-21

**Authors:** See Meng Lim, Jocelyn M. Choo, Hui Li, Rebecca O’Rielly, John Carragher, Geraint B. Rogers, Iain Searle, Sarah A. Robertson, Amanda J. Page, Beverly Muhlhausler

**Affiliations:** 1 School of Agriculture, Food and Wine, The University of Adelaide, Glen Osmond 5064, Australia; smlim@ukm.edu.my (S.M.L.); john.carragher@adelaide.edu.au (J.C.); 2 South Australian Health and Medical Research Institute, Adelaide 5000, Australia; jocelyn.choo@sahmri.com (J.M.C.); hui.li01@adelaide.edu.au (H.L.); rebecca.orielly@adelaide.edu.au (R.O.); geraint.rogers@sahmri.com (G.B.R.); amanda.page@adelaide.edu.au (A.J.P.); 3 Centre for Community Health Studies (ReaCH), Faculty of Health Sciences, Universiti Kebangsaan Malaysia, Jalan Raja Muda Abdul Aziz, Kuala Lumpur 50300, Malaysia; 4 College of Medicine and Public Health, Flinders University, Adelaide 5042, Australia; 5 Adelaide Medical School, The University of Adelaide, Adelaide 5000, Australia; sarah.robertson@adelaide.edu.au; 6 School of Biological Sciences, The University of Adelaide, Adelaide 5005, Australia; iain.searle@adelaide.edu.au; 7 Robinson Research Institute, The University of Adelaide, Adelaide 5000, Australia; 8 Commonwealth Scientific and Industrial Research Organisation, Adelaide 5000, Australia

**Keywords:** gastrointestinal health, gut microbiota, high amylose wheat, resistant starch

## Abstract

High amylose wheat (HAW) contains more resistant starch than standard amylose wheat (SAW) and may have beneficial effects on gastrointestinal health. However, it is currently unclear whether these effects differ according to the level of HAW included in the diet or between males and females. Male and female C57BL/6 mice (*n* = 8/group/sex) were fed SAW65 (65% SAW; control), HAW35 (35% HAW), HAW50 (50% HAW) or HAW65 (65% HAW) diet for eight weeks. Female but not male, mice consuming any amount of HAW exhibited accelerated gastric emptying compared to SAW65 group. In both sexes, relative colon weights were higher in the HAW65 group compared to SAW65 group and in females, relative weights of the small intestine and cecum were also higher in the HAW65 group. In females only, colonic expression of *Pyy* and *Ocln* mRNAs were higher in the HAW65 group compared to HAW35 and HAW50 groups. In both sexes, mice consuming higher amounts of HAW (HAW50 or HAW65) had increased fecal bacterial load and relative abundance of Bacteroidetes phylum and reduced relative abundance of Firmicutes compared to SAW65 group. These data are consistent with a beneficial impact of HAW on gastrointestinal health and indicate dose-dependent and sex-specific effects of HAW consumption.

## 1. Introduction

Wheat is one of the most widely consumed cereal grains in the world, with increasing global demand as a result of industrialization and westernization [1,2]. Wheat flour has unique properties and can be processed into a wide range of food products, including bread, noodles, biscuits and breakfast cereals, many of which are dietary staples. Consumption of these wheat-based products makes a major contribution to daily energy intake in many populations worldwide [1]. However, due to cultural and textural preferences, wheat is most commonly consumed in a highly processed and refined form, where the bran and germ fractions have been removed. While refining the grain does not affect the starch content, the fiber content is significantly lower than in whole grains, since this is largely found in the bran layer of the wheat kernel [3].

The lower dietary fiber content of refined grains has significant implications for the health benefits of these products [4]. This is particularly relevant for gastrointestinal health, since dietary fiber plays a critical role in maintaining optimal functioning of the gastrointestinal tract; adequate fiber intake increases fecal weight, promotes regular laxation by accelerating the passage of food through the digestive tract and reduces the risk of gastrointestinal disorders [5]. While very high intakes of certain fibers may be associated with gastrointestinal complaints, including abdominal discomfort, bloating, flatulence and diarrhea, there is little evidence that dietary fiber from a variety of sources causes significant adverse effects in healthy individuals [6]. Furthermore, there is increasing evidence that the gut microbiota plays an important role in maintaining gastrointestinal health, with alterations to the gut microbiota composition linked with several gastrointestinal diseases, including irritable bowel syndrome and inflammatory bowel disease [7]. Resistant starch is defined as a subgroup of dietary fiber that resists digestion in the upper gastrointestinal tract and reaches the colon intact. Fermentation of resistant starch and other highly fermentable fibers, including pectin, gum and mucilages, by colonic bacteria, results in an alteration in the gut microbiota composition and in the production of metabolites, which have been shown to act as signaling molecules for gut hormone release and gastrointestinal function [8]. Furthermore, the molecular weight of dietary fiber can influence the fermentability rate, with a low-molecular weight fiber rapidly fermented in the proximal colon [9]. While certain compounds, such as phytates, oxalates and tannins, may reduce mineral absorption, fermentation of certain fibers may also help to increase uptake of minerals in colon, including calcium and magnesium [10].

Despite this, dietary fiber intake in many populations worldwide remains well-below recommended levels [11,12]. In Australia, ~60% of children and >70% of adults in the 2011–2012 National Nutrition and Physical Activity Survey did not meet the recommended adequate daily intake of dietary fiber (children: 14–28 g; women: 25 g and men: 30 g) [11]. Therefore, there is a need for new approaches to sustainably increase fiber intake at the population level. Increasing the fiber content of staple foods, such as wheat-based products, offers an attractive solution.

High amylose wheat (HAW) is a novel wheat variety which has up to three times more amylose compared to standard wheat varieties [13]. This high amylose content is associated with higher levels of dietary fiber, mainly in the form of resistant starch, with HAW varieties having a resistant starch content as high as 11.2% compared to negligible amounts (<1%) in standard wheat [14]. In order to achieve optimal health benefits and ensure adequate micronutrient intakes, it will be important for individuals to include HAW-based products, with their high resistant starch content, as part of a balanced diet. It should also be noted, however, that HAW consumption has the potential to assist individuals in improving their micronutrient intakes, since HAW consists of higher levels of vitamin and mineral compared to standard wheat varieties [15]. The nutritional attributes of HAW underpin the hypothesis that consuming greater amounts of this wheat variety would be associated with improvements in gastrointestinal health [16]. Previous studies conducted in male Sprague-Dawley rats have reported that consuming a diet containing ~50% HAW (*w*/*w*) for 2–11 weeks improved indicators of gastrointestinal health in rats, as assessed by an increase in the weight of the large bowel, elevated concentrations of short-chain fatty acids in the digesta and reduced cecal pH [17,18].

In spite of these encouraging findings, the effects of increased dietary intake of HAW on functional measures of gastrointestinal health and gut microbiota composition and how these are influenced by the level of HAW in the diet, remain unknown. In addition, whether the effects differ between males and females is also unclear, since to our knowledge all previous studies using animal models to investigate the physiological effects of a HAW diet have been conducted exclusively in males [17,18,19]. Therefore, the aim of this study was to determine the effect of replacing standard amylose wheat (SAW) flour in the diet with increasing levels of HAW on gastrointestinal health parameters and gut microbiota composition in male and female mice.

## 2. Materials and Methods

### 2.1. Animals and Dietary Intervention

Details of the study design and dietary intervention have been described previously [20]. Briefly, eight-week-old C57BL/6 male and female mice (*n* = 64) were maintained in a temperature-controlled environment (22 ± 2 °C) under a 12-hr light-dark cycle at the South Australian Health and Medical Research Institute (SAHMRI) specific pathogen-free and PC2 animal facility (Adelaide, SA, Australia) with *ad libitum* access to food and purified water. Mice of the same sex were housed in groups of four in ventilated cages (GM500 Mouse IVC Green Line, Tecniplast, Buguggiate, VA, Italy), which contained dust-free laboratory bedding and enrichment (nesting material and rodent tunnels).

The animals were randomly allocated to one of four dietary groups (*n* = 8/group/sex): the SAW65 group were fed a diet containing 650 g/kg SAW (65%); the HAW35 group were fed a diet containing 350 g/kg HAW (35%) and 300 g/kg SAW (30%); the HAW50 group were fed a diet containing 500 g/kg HAW (50%) and 150 g/kg SAW (15%); and the HAW65 group were fed a diet containing 650 g/kg HAW (65%). Table 1 shows the composition and source of ingredients for each experimental diet. The contents of total dietary fiber and resistant starch for each diet are: SAW65: 13.5% and 1.2%; HAW35: 16.2% and 1.6%; HAW50: 17.3% and 1.7%; and HAW65: 18.5% and 1.9%, respectively. Detailed information on the production of the experimental diets has been published previously [20]. Briefly, all diets were made in-house to the AIN-93M formulation with the exception that a portion of the carbohydrate component was replaced by whole grain flour of SAW and/or HAW in the proportions indicated above. In order to mimic how humans typically consume grains, the wheat flour was first cooked for an hour at 70 °C and 80% humidity to promote gelatinization, followed by storage at 4 °C overnight to promote retrogradation for resistant starch formation. The HAW used in this study contained ~46% of amylose in its total starch content and was developed through a normal selective breeding process [20]. All animal procedures were approved by the SAHMRI Animal Ethics Committee (Project code: SAM294) and were in compliance with the Australian National Health and Medical Research Council’s code for the care and use of animals for scientific purposes (8th edition 2013) and South Australia Animal Welfare Act 1985.

### 2.2. Assessment of Gastric Emptying Rate

After seven weeks of consuming the experimental diets, all mice were assessed for gastric emptying via a gastric emptying breath testing, using previously described methods [21,22]. Briefly, mice were fasted overnight in individual cages with wire mesh at the base to restrict coprophagy. On the following morning, mice were placed in individual air-tight containers with the lids of the containers removed between sampling to supply oxygen. After a baseline breath sample collection, the mice were given 0.1 g of baked egg yolk containing 1 μL/g of ^13^C-labelled octanoic acid (99% enrichment, Cambridge Isotope Laboratories, Andover, MA, USA), which consumed within 1 min. Breath samples were then collected at 5 min intervals for the first 30 min after food consumption followed by every 15 min until 150 min. After this, the mice were returned to their home cages with free access to water and their respective diets. The ^13^CO_2_ content of breath samples was analyzed with an isotope ratio mass spectrometer (ABCA 20/20 Europa Scientific, Crewe, Cheshire, UK). The measured values were presented as ∆^13^CO_2_ (‰) and area under the curve (AUC; ‰ x min) as described by Uchida and colleagues [23].

### 2.3. Post-Mortem and Tissue Collection

After eight weeks on their respective experimental diets, mice were individually housed in cages with wire-mesh bases, to prevent coprophagy and fasted overnight. On the following morning, mice were anaesthetized using isoflurane inhalation (5% induction, 2–3% maintenance) and blood samples were collected from the abdominal aorta. Blood samples were immediately centrifuged (1000× *g*, 15 min at 4 °C) and plasma was snap-frozen in liquid nitrogen (N_2_) and stored at −80 °C until analysis. Following blood collection, mice were euthanized by cervical dislocation. Gastrointestinal tract organs, including the stomach, small intestine, cecum and colon were collected, weighed, snap-frozen in liquid N_2_ and stored at −80 °C until analysis. Food matter and fecal pellets were not specifically removed as the overnight fast ensured that virtually all of the digesta content of the animals was either absorbed or excreted out of the gastrointestinal tract. Cecal content was also collected, snap-frozen in liquid N_2_ and stored at −80 °C until analysis. All collections were carried out within 1–2 h from the beginning of light phase and the majority of female mice were sampled during either the diestrus (72%) or metestrus phase (19%) of the estrus cycle. All mice were monitored daily for their health status and no signs of ill health were noted in any of the mice during the experimental period. The gastrointestinal organ weights were expressed as relative organ weight, which was calculated by dividing organ weight by the total body weight of the animal.

### 2.4. Plasma Hormone Concentrations

The plasma gastric inhibitory peptide (GIP) concentrations were determined, in duplicate, using the Milliplex xMAP Luminex Assay according to the manufacturer’s instructions (Catalogue No.: MMHE-44K; Merck Millipore, Temecula, CA, USA). The procedures of the assay were followed strictly according to the manufacturer’s instructions and all plasma samples were evaluated neat (40 μL). The minimum detection limit for GIP was 0.001 ng/mL and the intra-assay coefficient of variation was 8.43%.

### 2.5. Measurement of Cecal Content pH

Thawed cecal contents (0.3 g) were homogenized in 0.3 mL of ultrapure water (Milli-Q, Millipore, Bedford, MA, USA) by vortexing and the pH was measured, in duplicate, using a pH meter (Eutech Instruments pH 510, Singapore) that had been calibrated at room temperature with buffer solutions at pH 4 and pH 7.

### 2.6. Reverse Transcription-Quantitative Polymerase Chain Reaction (RT-qPCR)

Total RNA was extracted from 30 mg of cecum and colon tissues, respectively, using a RNeasy^®^ Mini kit according to the manufacturer’s instructions (QIAGEN, Hilden, Germany). The RNA quantity and quality were measured with a Nanodrop spectrophotometer (Thermo Fisher Scientific, Madison, WI, USA). RNA samples with a A260/A280 ratio of 1.9 to 2.1 were considered of acceptable quality. A total of 25 ng/µL of RNA was reverse transcribed and expression of target and housekeeper genes was quantified, in duplicate, using an EXPRESS One-Step SuperScript^®^ qRT-PCR Universal Kit (Invitrogen, Life Technologies, Carlsbad, CA, USA) and pre-designed TaqMan gene expression probes (Applied Biosystems, Thermo Fisher Scientific, Pleasanton, CA, USA) on the QuantStudio^TM^ 7 Flex real-time PCR System (Applied Biosystems, Thermo Fisher Scientific). The following cycling conditions were used: 50 °C for 15 min, 95 °C for 2 min, followed by 40 cycles of 95 °C for 15 s and 60 °C for 15 min. TaqMan probes were used to detect mRNAs encoding peptide YY (*Pyy*; Mm00520715_m1), proglucagon (*Gcg*; Mm01269055_m1), occludin (*Ocln*; Mm00500912_m1), mucin 2 (*Muc2*; Mm01276696_m1), hypoxanthine guanine phosphoribosyl transferase (*Hprt*; Mm01545399_m1), beta-2 microglobulin (*B2m*; Mm00437762_m1) and peptidylprolyl isomerase A (*Ppia*; Mm02342429_g1). The NormFinder (https://moma.dk/normfinder-software) stability value was calculated and indicated that *Hprt* and *B2m* provided appropriate stable housekeeper genes for both cecum (0.14) and colon (0.07) samples. Negative controls, containing all reagents but with water instead of RNA, were included for each probe on each plate to confirm the absence of contamination. Data were analyzed with a QuantStudio^TM^ real-time PCR System Software v.1.3 (Applied Biosystems, Thermo Fisher Scientific, Waltham, MA, USA). Cycle threshold (Ct) values of the target genes were normalized to the housekeeper genes and the relative gene expression was calculated using the 2^−ΔΔCt^ method [24].

### 2.7. Fecal Collection, DNA Extraction and Bacterial Quantification

Fresh fecal pellets were collected between 10–11 a.m. with sterile toothpicks and placed into sterile 1.5 mL Eppendorf tubes and then stored at −80 °C. At the time of DNA extraction, fecal pellets were weighed, resuspended in 300 µL of phosphate-buffered saline (pH 7.2) by vortexing and pelleted by centrifugation at 10,000× *g* for 10 min. The supernatant was stored at −80 °C and the pellet underwent DNA extraction using the QIAGEN PowerLyzer PowerSoil kit (QIAGEN, Hilden, Germany), as described previously [25]. Total bacterial load was quantified based on the 16S rRNA abundance by using a SYBR^®^ PCR assay, as described previously [26]. Briefly, each reaction comprised of 1X SYBR^®^ Green, 0.2 µM of each forward and reverse primer and 1 µL of DNA in a 35 µL total reaction volume. Each reaction was aliquoted into three technical replicates with a volume of 10 µL. The number of 16S rRNA gene copies was normalized against the fecal weight (mg) of each sample.

### 2.8. 16S rRNA Gene Amplicon Sequencing and Bioinformatics Analysis

Barcoded libraries of the bacterial 16S rRNA gene V4 hypervariable region were prepared by using fecal DNA extracts and sequencing was performed on an Illumina MiSeq platform at the David R Gunn Genomics Facility, SAHMRI (Adelaide, SA, Australia). Briefly, amplicons were generated using the modified universal bacterial primer pairs 515F (5′-TCGTCGGCAGCGTCAGATGTGTATAAGAGCAGGTGCCAGCMGCCGCGGTAA-3′) and 806R (5′-GTCTCGTGGGCTCGGAGATGTGTATAAGAGACAGGGACTACHVGGGTWTCTAAT-3′) with Illumina adapter overhang sequences (indicated by underline), according to the Illumina MiSeq 16S Metagenomic Sequencing Library Preparation protocol with minor modifications, as described previously [25]. Raw sequence files are publicly available from the Sequence Read Archive (SRA) database (Bioproject ID: PRJNA630838).

**Figure A1:**
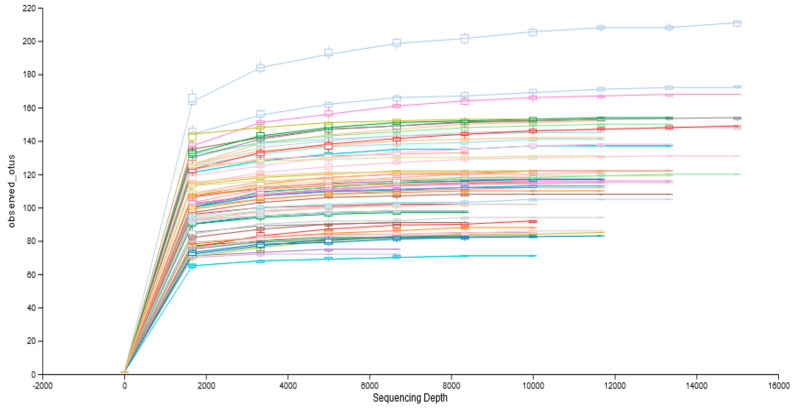
Rarefaction curve sequences of the fecal microbial community of all samples. The rarefaction curve indicates the number of operational taxonomic units (OTU) observed for each sample and were generated using the QIIME2 tool.

Bioinformatics analyses of paired-end 16S rRNA V4 sequence reads were conducted using the Quantitative Insights Into Microbial Ecology (QIIME) software (v2.2019.4) [27]. Denoising was performed using Dada2 and taxonomic classification of amplicon sequence variants were performed against the SILVA (v132) reference database, based on 16S rRNA V4 hypervariable region of operational taxonomic units that were clustered at 97% similarity. Spurious amplicon sequence variants, including those assigned as mitochondria and chloroplast, were removed. All samples were sub-sampled to the lowest sample read depth of 7285 sequence reads, at which the rarefaction curve of observed species had reached an asymptote (Figure A1).

### 2.9. Statistical Analysis

Statistical analysis for functional gastrointestinal measures and AUC calculation were performed separately for each sex by using the GraphPad Prism software (version 7.04, San Diego, CA, USA). Differences between groups were assessed using one-way ANOVA and Tukey’s post-hoc analysis where appropriate. Analysis of the gut microbiota was performed separately by sex, as well as in combined data from males and females. Normally distributed microbiota data were analyzed using ANOVA with sex and diet as the factors and Tukey’s post-hoc tests. Non-parametric microbiota data were analyzed using Kruskal-Wallis with the Dunn’s test for post-hoc analysis. Microbiota composition was analyzed using permutational ANOVA (PERMANOVA) based on the weighted Unifrac distances of samples [28,29]. Adjusted *p*-values were obtained using the false discovery rate (FDR) method. Significance was considered as *p* < 0.05.

## 3. Results

### 3.1. Gastric Emptying Rate

**Figure 1:**
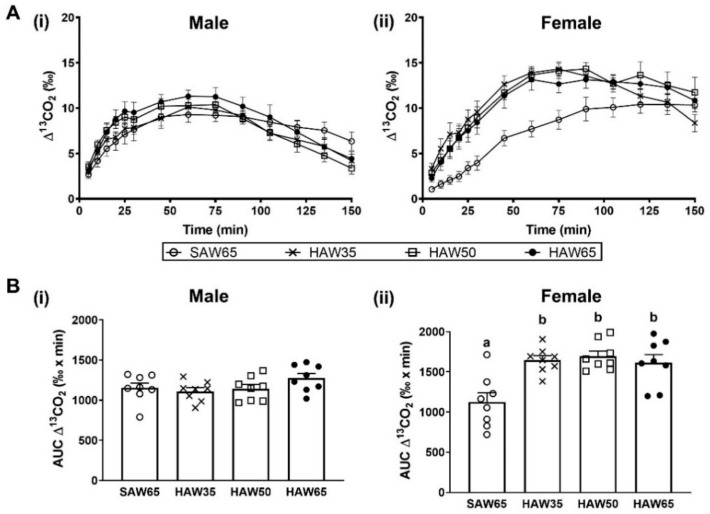
Gastric emptying rate expressed as (**A**) ^13^CO_2_ excretion curve and (**B**) area under the curve (AUC) in (**i**) male and (**ii**) female mice fed diets containing 65% standard amylose wheat (SAW65), 30% SAW and 35% high amylose wheat (HAW35), 15% SAW and 50% HAW (HAW50) or 65% HAW (HAW65) as determined by ^13^C-labelled octanoic breath test after seven weeks of feeding. Data are shown as mean ± SEM (*n* = 8 mice/group). Values with different superscripts indicate significant differences between groups (*p* < 0.05) as determined by one-way ANOVA and Tukey’s post-hoc tests.

In males, there were no significant differences in gastric emptying rates between the diet groups (Figure 1A(i),B(i)). In females, the rates of gastric emptying were increased in mice consuming diets containing any level of HAW when compared to those consuming the standard SAW65 diet, as reflected by the elevated ^13^CO_2_ excretion value (Figure 1A(ii)) as well as the increased AUC for the ^13^CO_2_ excretion curves (*p* < 0.05; Figure 1B(ii)).

### 3.2. Body Weights

The initial and end-point body weights of all mice have been previously published [20]. Briefly, there was no difference in the initial body weights between the groups in either male or female mice. However, at the end of the feeding period, male mice consuming any level of HAW were heavier compared to the SAW65 group. In female mice, however, there was no difference in body weight between dietary groups at the end of the feeding period.

### 3.3. Gastrointestinal Tract Organ Weights, pH of Cecal Content and Plasma GIP Concentrations

In males, relative weights of the small intestine were lower in mice consuming the HAW35 diet when compared to all other diet groups (*p* < 0.05; Table 2). Relative colon weights in male mice consuming the HAW65 diet were significantly higher than in males that consumed either the SAW65 (*p* < 0.01) or HAW35 (*p* < 0.01) diet but relative weights of the stomach or cecum were similar between groups.

In female mice, the relative weights of the small intestine were higher following consumption of the HAW65 diet compared to the SAW65 (*p* < 0.01) or HAW35 (*p* = 0.02) diet. Female mice that consumed either the HAW65 or HAW50 diet also had significantly higher relative cecum and colon weights compared to the SAW65 group, however relative stomach weights were not different between diet groups.

There were no differences in the pH of the cecal content or plasma GIP concentrations between diet groups in either male or female mice (Table 2).

### 3.4. Cecum and Colon Gene Expression

In the cecum of males, expression of *Pyy* mRNA was lower in mice who consumed the HAW35 diet when compared to the SAW65 diet (*p* = 0.02) whereas *Pyy* mRNA expression in the colon was not different between groups. There were also no differences in mRNA expression of either *Gcg* (that encodes preproglucagon) or the gut barrier markers, *Ocln* and *Muc2*, in either cecal or colonic tissues between diet groups (Table 3).

In females, *Pyy* mRNA expression in the colon but not in cecum, was higher in mice consuming the HAW65 diet when compared to those consuming the HAW50 diet (*p* = 0.01; Table 3) but not the HAW35 or SAW65 diets. Similarly, in the colon but not the cecum, *Ocln* mRNA expression was higher in those mice that consumed the HAW65 diet when compared to those mice consuming the HAW35 diet (*p* = 0.02). There were no differences in the expression of either *Gcg* or *Muc2* mRNA between diet groups in either the cecum or colon.

### 3.5. Gut Bacterial Load and Microbiota Diversity

**Figure 2:**
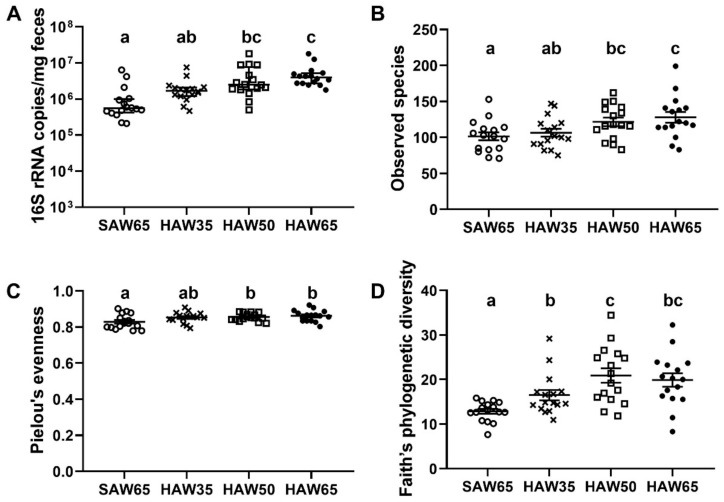
(**A**) Total bacterial load and (**B**) alpha diversity measures of microbial richness (observed species), (**C**) evenness (Pielou’s evenness) and (**D**) diversity (Faith’s phylogenetic diversity) in mice (both sexes combined) fed diets containing 65% standard amylose wheat (SAW65), 30% SAW and 35% high amylose wheat (HAW35), 15% SAW and 50% HAW (HAW50) or 65% HAW (HAW65) after eight weeks of feeding. Data are shown as median ± IQR for non-parametric data (total bacterial load) or mean ± SEM for parametric data (alpha diversity analyses; *n* = 16 mice/group). Values with different superscripts indicate significant differences between groups (*p* < 0.05) as determined by Kruskal-Wallis and Dunn’s post-hoc tests or ANOVA and pairwise comparisons with false discovery rate correction for *p*-values.

**Figure A2:**
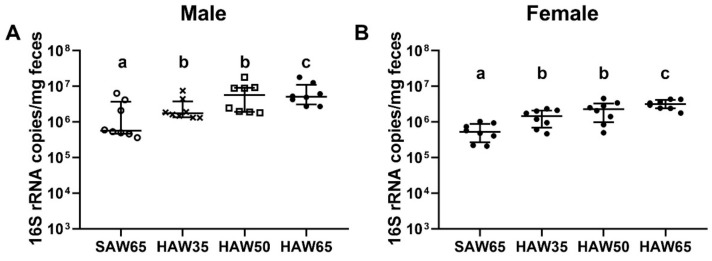
Total bacterial load in (**A**) male or (**B**) female mice fed diets containing 65% standard amylose wheat (SAW65), 30% SAW and 35% high amylose wheat (HAW35), 15% SAW and 50% HAW (HAW50) or 65% HAW (HAW65) after eight weeks of feeding. Data are shown as median ± IQR (*n* = 8 mice/group/sex). Values with different superscripts indicate significant differences between groups (*p* < 0.05) as determined by Kruskal-Wallis and Dunn’s post-hoc tests.

Mice consuming either the HAW50 or HAW65 diet had a higher fecal bacterial load when compared to those consuming the SAW65 diet, in both overall (*p* < 0.01; Figure 2A) and when males (*p* < 0.05; Figure A2A) and females (*p* < 0.05; Figure A2B) were separated in the analysis. Further, mice consuming the highest level of HAW (HAW65) also exhibited a higher fecal bacterial load compared to mice consuming the diet containing the lowest level of HAW (HAW35) in both male and female mice.

**Figure A3:**
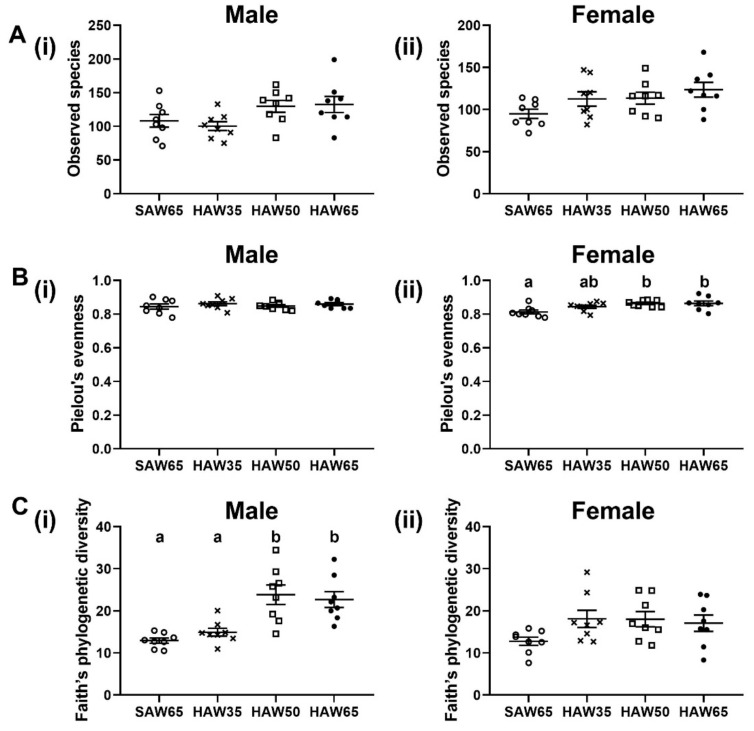
Alpha diversity measures of microbial (**A**) richness (observed species), (**B**) evenness (Pielou’s evenness) and (**C**) diversity (Faith’s phylogenetic diversity) in (**i**) male or (**ii**) female fed diets containing 65% standard amylose wheat (SAW65), 30% SAW and 35% high amylose wheat (HAW35), 15% SAW and 50% HAW (HAW50) or 65% HAW (HAW65) after eight weeks of feeding. Data are shown as mean ± SEM (*n* = 8 mice/group/sex). Values with different superscripts indicate significant differences between groups (*p* < 0.05) as determined by ANOVA and pairwise comparisons tests with false discovery rate correction for *p*-values.

Mice consuming the diet containing the two highest levels of HAW (HAW50 or HAW65) also exhibited higher alpha diversity measures of microbial richness (observed species), evenness (Pielou’s evenness) and diversity (Faith’s phylogenetic diversity) when compared to those consuming the SAW65 diet (FDR *p* < 0.05, Figure 2B–D). In addition, mice consuming the lowest level of HAW (HAW35) had a lower gut microbial richness when compared to the HAW65 diet (FDR *p* = 0.045, Figure 2B) and a lower gut microbial diversity when compared to HAW50 diet (FDR *p* = 0.021, Figure 2D). When analyses were conducted separately for each sex, there were no differences between diet groups in gut microbial richness (Figure A3A(i)) or gut microbial evenness (Figure A3B(i)) in male mice. However, male mice consuming either the HAW50 or HAW65 diet had a higher gut microbial diversity when compared to those consuming the SAW65 diet (FDR *p* < 0.001, Figure A3C(i)). In females, mice consuming either the HAW50 or HAW65 diet had a higher gut microbial evenness when compared to those consuming the SAW65 diet (FDR *p* < 0.05, Figure A3B(ii)). There were, however, no differences in either gut microbial richness (Figure A3A(ii)) or gut microbial diversity (Figure A3C(ii)) between diet groups.

### 3.6. Gut Microbiota Composition

Analysis of fecal microbiota composition based on weighted Unifrac distances indicated compositional differences between the diet groups (*p* = 0.021, square root of estimates of components of variation (ECV) = 0.043), with no differences between sexes (*p* = 0.324, square root ECV = 0.0065) or sex by diet interaction (*p* = 0.929, square root ECV = −0.0289). At the phylum level, differences between the diet groups were observed in the relative abundances of the two most abundant phyla, Bacteroidetes and Firmicutes (*p* < 0.0001 for both phyla) but not for the other phyla, including Proteobacteria, Actinobacteria, Patescibacteria, Tenericutes and Verrucomicrobia (*p* > 0.05; Table 4). Specifically, mice consuming diets containing any levels of HAW (35–65% HAW) showed higher relative abundance of Bacteroidetes but reduced relative abundance of Firmicutes, in comparison to those consuming the SAW65 diet. These changes were reflected in a lower Firmicutes:Bacteroidetes ratio in mice consuming any level of HAW compared to those consuming the SAW65 diet (*p* < 0.001). These differences in the relative abundances of Bacteroidetes and Firmicutes and Firmicutes:Bacteroidetes ratio between the diet groups were also observed for both males and females when the analyses were conducted separately in each sex (Table 4).

**Figure 3:**
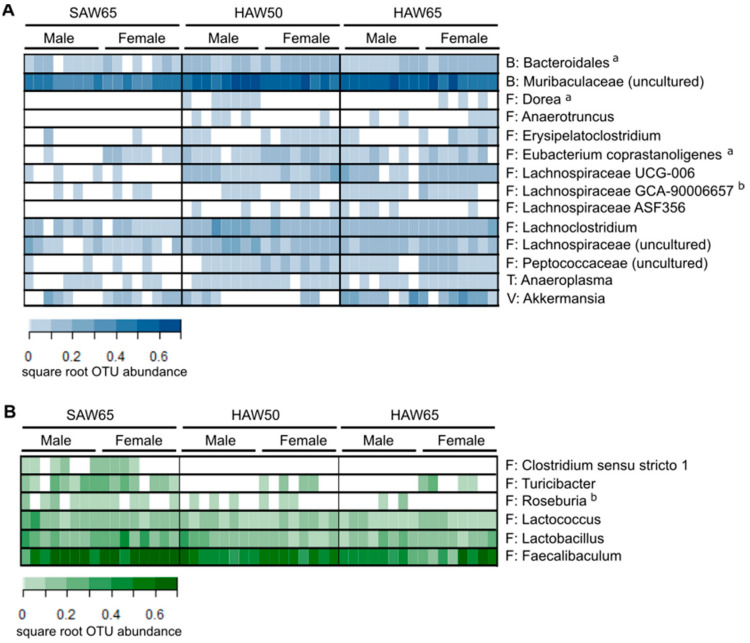
Heatmap of bacterial genera, which relative abundance significantly (**A**) increased or (**B**) decreased in mice fed diets containing 15% standard amylose wheat (SAW) and 50% high amylose wheat (HAW; HAW50) or 65% HAW (HAW65), in comparison to those consuming diet containing 65% SAW (SAW65) after eight weeks of feeding. Relative abundances of each taxa in male or female mice are based on square root relative abundances (*n* = 8 mice/group/sex). Values with different superscripts indicate significant differences between groups (*p* < 0.05) as determined by Mann-Whitney tests and pairwise comparisons with false discovery rate correction for *p*-values. Taxa that were only significant difference between HAW50 and SAW65 or HAW65 and SAW65, are denoted with the superscript “a” or “b,” respectively. The phyla Bacteroidetes (B), Firmicutes (F), Tenericutes (T) and Verrucomicrobia (V) are denoted to indicate the phylum group of bacterial taxa that were significantly altered.

Pairwise compositional analyses performed between the diet groups indicated that the gut microbiota composition of mice consuming the SAW65 diet differed to that of mice consuming either the HAW50 (*p* = 0.029) or HAW65 (*p* = 0.043) diets. At the genera level, the abundance of nine bacterial taxa, predominantly of the Firmicutes phylum, as well as the Bacteroidetes and Tenericutes phyla, were increased in both the HAW50 and HAW65 diet groups compared to the SAW65 diet group (FDR *p* < 0.05; Figure 3). Taxa that were decreased in both the HAW50 and HAW65 diet groups, compared to the SAW65 diet group, were *Clostridium sensu stricto 1*, *Turicibacter*, *Lactococcus*, *Lactobacillus* and *Faecalibaculum*, all of which are under the Firmicutes phylum (FDR *p* < 0.05). The taxa *Dorea*, *Eubacterium coprastanoligenes* and a taxon under the Bacteroidales order increased only in the HAW50 diet group compared to the SAW65 diet group, whereas *Lachnospiraceae GCA-90006657* and *Akkermansia* were increased and *Roseburia* were decreased in the HAW65 diet group compared to the SAW65 diet group (Figure 3).

## 4. Discussion

This study demonstrates that a diet containing HAW at levels between 35% and 65% of total diet weight exhibited dose-dependent and sex-specific effects on a number of gastrointestinal parameters, including increasing the rate of gastric emptying, increasing the expression of *Pyy* and *Ocln* and producing significant shifts in gut microbiota composition. The changes in gastrointestinal function and gene expression tended to be more pronounced in female mice and microbiota changes were present at higher levels of HAW consumption, suggesting that these factors are important to consider when interpreting and extrapolating these findings.

Our finding that female mice consuming any level of HAW exhibited an accelerated rate of gastric emptying compared to those consuming the control diet (SAW65) was unexpected given the higher dietary fiber content in the HAW diets [20]. Foods rich in dietary fiber, especially soluble fiber, have been reported in several previous studies to slow gastric emptying rate [30,31,32]. This is largely a result of fiber’s high water-holding capacity and viscosity, both of which increase gastrointestinal fullness and limit the ability of food particles to enter the duodenum [33,34]. However, not all studies have reported such effects [35,36]; one study reported that consumption of 15 g of bran twice daily for four days had no effect on gastric emptying rate in healthy human subjects [35], while other studies reported that healthy human subjects who consumed high fiber products, containing either 5% cellulose or low (2.5%) or high (5%) doses of alginate or a type 3 resistant starch (retrograded amylose) exhibited accelerated gastric emptying rates [36,37], consistent with our findings. Additional research suggests that the impact of dietary fiber on gastric emptying is also dependent on the type, amount and hydration state of the dietary fiber consumed [38], which may account for the variability in results. Enhanced gastric emptying rates would be expected to be associated with a shorter transit time through the gut. This, in turn, would reduce digestion and absorption of macronutrients, including fat and could potentially reduce energy uptake and limit fat deposition. This hypothesis is at least partially supported by our previous finding that female mice consuming diets containing between 35–65% HAW, tended to have lower relative total and gonadal fat mass [20].

The higher relative weights of the small intestine, cecum and colon in female mice consuming HAW diets were consistent with a previous study in which male Sprague-Dawley rats fed a 48% high amylose maize diet (*w*/*w*) for 4 weeks exhibited higher small intestine, cecum and colon weights and had a higher daily fecal output compared to controls [39]. Interestingly, however, in the males in the current study, only the relative weight of the colon was higher in those consuming the highest level of HAW diet (HAW65) when compared to both SAW65 and HAW35 groups, suggesting that effects of our HAW diet on gastrointestinal organ weight were more pronounced in females. It is possible that the increased gastrointestinal organ weights in animals consuming the HAW diets, were due to the increased proliferation of the intestinal epithelial cells, likely stimulated by the fermentation products of dietary fiber, including short-chain fatty acids [40]. This may also explain the greater effects in females compared to males, since males have a reduced colon transit time and thus absorb less short-chain fatty acids than females [41,42] but this is highly speculative and requires further investigation. Irrespective of the underlying mechanism, however, higher gastrointestinal organ weight is likely to be associated with improved gastrointestinal health, since an increased thickness of the intestinal epithelium can reduce gut atrophy and may enhance nutrient absorption in the small intestine [43,44].

Diets containing higher amounts of fermentable fiber are typically associated with increased production of short-chain fatty acids, which in turn reduces luminal pH. Given the higher fiber content of the HAW compared to the SAW diets, the lack of a difference in the pH of the cecal content between dietary groups was therefore unexpected. Our finding is, however, consistent with a previous study [17] which also found no difference in cecal content pH in male Sprague-Dawley rats consuming a Western style diet supplemented with either HAW or SAW for 11 weeks. In contrast, another study reported that consuming a HAW diet for two weeks significantly reduced cecal pH in young male Sprague-Dawley rats [18]. However, the level of amylose in the HAW variety used in this previous study was much higher than in the current study (~70% vs. ~46%) and this may account for the different results. It is therefore possible that a higher amylose level than occurs in the HAW variety used in our study, which would be expected to result in greater increases in resistant starch content, is required to promote fermentation to a level that is sufficient to reduce luminal pH.

Female mice consuming the diet containing the highest level of HAW (HAW65) exhibited a higher colonic expression of *Pyy* when compared to those consuming a lower level (HAW50). Pyy plays a major role in maintaining energy balance and appetite, influences gut motility, gastrointestinal cell differentiation and proliferation [45] and increased *Pyy* treatment for two weeks has been associated with improvements in several structural markers of gastrointestinal health [46]. Our finding of increased *Pyy* expression is consistent with findings in another study, in which feeding a higher level of resistant starch from Hi-Maize corn starch was associated with increased expression of both *Pyy* and *Gcg* in the colonic epithelial cells in male Sprague-Dawley rats [47]. In response to the Hi-Maize corn starch, however, the increased mRNA expression extended to the cecum [47], possibly due to the higher level of resistant starch in the Hi-Maize corn starch.

The mRNA expression of *Muc2*, the major secretory protein secreted by intestinal goblet cells, was not influenced by consumption of the HAW diet. This is in agreement with the results of a previous study by Hedemann and co-workers who found that *Muc2* mRNA expression in the cecum and colon was not different in male Wistar rats that consumed different sources of carbohydrates, including cellulose, pectin, inulin, resistant starch or barley hulls [48]. The maintenance of *Muc2* mRNA expression is important, since the mucus layer acts as an important barrier protecting gut epithelial cells from damage and infection by pathogenic bacteria [49] and reduced *Muc2* mRNA has been associated with several gastrointestinal diseases [50]. While the consumption of the HAW diets did not appear to influence *Muc2* expression, female mice consuming the HAW65 diet had increased *Ocln* abundance when compared to those consuming the HAW35 diet. Occludin is a tight junction protein crucial for maintaining intestinal barrier integrity and impairments in the intestinal epithelial barrier increase the risk of numerous disorders, including metabolic endotoxemia, inflammatory bowel and celiac diseases [51,52]. In addition, a randomized placebo-controlled pilot study (*n* = 34) has also reported that supplementation of a gluten-free diet with prebiotic oligofructose-enriched inulin (10 g per day) increased the concentrations of intestinal barrier integrity marker, zonulin, in children with celiac disease [53]. It therefore appears that a high HAW diet may have some beneficial effects on intestinal barrier integrity in female mice, however, this requires functional verification. Overall, the findings of this study suggest that the effect of HAW consumption on gastrointestinal gene expression is gene-specific and may vary in different regions of the gastrointestinal tract.

In this study, we demonstrated that consumption of the higher levels of HAW, HAW50 or HAW65, was associated with higher fecal bacterial load and alpha diversity of microbiota, including richness, evenness and diversity, suggesting that increased consumption of HAW promotes fecal bacterial growth. This is in line with previous studies, in which higher fecal bacterial load and bacterial diversity are observed when animals are fed diets supplemented with either soluble or fermentable fiber [54,55]. Furthermore, we found that consumption of the higher levels of HAW, induced a shift in the composition of the gut microbiota towards an increased relative abundance of the Bacteroidetes phylum and decreased relative abundance of the Firmicutes phylum, ultimately reducing the Firmicutes:Bacteroidetes ratio. This may due to the higher fiber content of the HAW favoring the growth of members of the Bacteroidetes phylum, since fiber is known to be a substrate that favors the growth of these bacteria [56]. The lower Firmicutes:Bacteroidetes ratio may be a marker of improved gastrointestinal health, since previous studies have reported an increased ratio of Firmicutes to Bacteroidetes in patients with either irritable bowel syndrome [57] or inflammatory bowel diseases [58]. An increased ratio of Firmicutes: Bacteroidetes, also increases the efficiency of energy absorption and has been associated with obesity in both humans [59] and mice [60]. Thus, the lower Firmicutes:Bacteroidetes ratio may also contribute to the lower adiposity we observed in mice consuming the HAW diet in our previous study [20].

The beneficial effects of HAW diets on the microbiota were further supported by the increased relative abundance of several bacteria, including Bacteroidales, Muribaculaceae, Lachnospiraceae and *Anaeroplasma* and reduced relative abundance of bacteria, including *Clostridium sensu stricto 1* and *Turicibacter* in mice consuming diets containing the higher levels of HAW. Members of the order *Bacteroidales*, have been shown to produce antimicrobial toxins, which may limit the growth of opportunistic pathogens [61]. Several taxa of the *Muribaculaceae* [62] and *Lachnospiraceae* families [63], are capable of fermenting plant polysaccharides to produce short-chain fatty acids, which serve as energy sources for the colonic epithelium and maintenance of the gut mucosal barrier [64], while *Anaeroplasma* contribute to improving the integrity of the intestinal barrier [65,66]. Conversely, overgrowth of *Clostridium sensu stricto 1* and *Turicibacter* are associated with increased frequency of diarrhea [67], risks of necrotic enteritis [68], acute appendicitis [69] and ulcerative colitis [70].

## 5. Conclusions

In conclusion, we have demonstrated that increased consumption of HAW has effects on gastrointestinal weight, gene expression and function, including enhanced gastric motility and increased mRNA expression of the intestinal barrier marker *Ocln* and the gut hormone *Pyy*, particularly in female mice that consumed diets with higher levels of HAW. Furthermore, increased consumption of HAW was associated with increased fecal bacteria load and altered composition of the gut microbiota in both male and female mice in a manner consistent with favorable effects on gut health, particularly at higher levels of HAW intakes. Together, these findings add to the existing evidence that HAW may has beneficial effects for maintaining and improving gastrointestinal health. The findings also indicate that the impact of HAW on a number of gastrointestinal measures is dependent on both the sex of the animal and the level of inclusion in the diet, highlighting the importance of taking these factors into account in the design of future studies in this area.

## Figures and Tables

**Table 1:** Modified AIN-93M diets with different levels of wheat flour.

Ingredient (g/kg)	Source	SAW65	HAW35	HAW50	HAW65
Standard amylose wheat	Waite Research Institute (Glen Osmond, SA, Australia)	650.00	300.00	150.00	0.00
High amylose wheat	Waite Research Institute (Glen Osmond, SA, Australia)	0.00	350.00	500.00	650.00
Maltodextrin	Bulk Nutrients (Grove, TAS, Australia)	42.97	42.97	42.97	42.97
Sucrose	Sigma (Saint Louis, MO, USA)	27.72	27.72	27.72	27.72
Casein	Rogers & Company Foods Pty Ltd. (Hampton, VIC, Australia)	140.00	140.00	140.00	140.00
L-cystine	Sigma (Saint Louis, MO, USA)	1.80	1.80	1.80	1.80
Soybean oil	Chia Khim Lee Food Industries Pte Ltd. (Defu Lane, Singapore)	40.00	40.00	40.00	40.00
Cellulose	Sigma (Saint Louis, MO, USA)	50.00	50.00	50.00	50.00
Mineral mix, AIN-93M-MX	MP Biomedicals (Solon, OH, USA)	35.00	35.00	35.00	35.00
Vitamin mix, AIN-93-VX	MP Biomedicals (Solon, OH, USA)	10.00	10.00	10.00	10.00
Choline bitartrate	Sigma (Saint Louis, MO, USA)	2.50	2.50	2.50	2.50
TBHQ, antioxidant	Sigma (Saint Louis, MO, USA)	0.008	0.008	0.008	0.008
Total		1000	1000	1000	1000

SAW, Standard amylose wheat; HAW, High amylose wheat.

**Table 2:** Relative gastrointestinal tract organ weights, cecal content pH and plasma gastric inhibitory peptide concentrations in male and female mice after eight-week feeding.

Parameter	SAW65	HAW35	HAW50	HAW65
**Male**				
Stomach (mg/g BW)	8.74 ± 1.00	7.82 ± 0.53	8.90 ± 0.65	8.25 ± 0.44
Small intestine (mg/g BW)	30.25 ± 0.72 ^b^	26.62 ± 0.67 ^a^	29.55 ± 0.96 ^b^	30.05 ± 0.60 ^b^
Cecum (mg/g BW)	8.47 ± 1.12	9.13 ± 0.92	7.98 ± 0.56	9.92 ± 1.03
Colon (mg/g BW)	5.80 ± 0.29 ^a^	5.83 ± 0.44 ^a^	6.64 ± 0.26 ^ab^	7.69 ± 0.21 ^b^
pH of cecum contents	8.07 ± 0.05	7.96 ± 0.09	7.98 ± 0.07	8.10 ± 0.07
Plasma gastric inhibitory peptide (ng/mL)	0.08 ± 0.01	0.08 ± 0.01	0.07 ± 0.01	0.06 ± 0.01
**Female**				
Stomach (mg/g BW)	9.87 ± 0.67	9.65 ± 0.53	9.03 ± 0.57	10.76 ± 0.42
Small intestine (mg/g BW)	34.53 ± 0.89 ^a^	36.24 ± 1.18 ^a^	37.05 ± 0.74 ^ab^	40.40 ± 0.81 ^b^
Cecum (mg/g BW)	8.05 ± 0.47 ^a^	9.11 ± 0.80 ^ab^	11.22 ± 0.46 ^b^	11.35 ± 0.92 ^b^
Colon (mg/g BW)	7.12 ± 0.39 ^a^	8.35 ± 0.57 ^ab^	9.90 ± 0.85 ^b^	10.08 ± 0.38 ^b^
pH of cecum contents	8.12 ± 0.04	8.00 ± 0.06	8.07 ± 0.07	7.98 ± 0.06
Plasma gastric inhibitory peptide (ng/mL)	0.08 ± 0.02	0.04 ± 0.01	0.06 ± 0.01	0.06 ± 0.01

Mice fed diets containing 65% standard amylose wheat (SAW65), 30% SAW and 35% high amylose wheat (HAW35), 15% SAW and 50% HAW (HAW50) or 65% HAW (HAW65). Data are shown as means ± SEM (*n* = 8 mice/group/sex). Values with different superscripts in a row indicate significant differences between groups (*p* < 0.05) as determined by one-way ANOVA and Tukey’s post-hoc test. BW, body weight.

**Table 3:** Relative cecal and colonic mRNA levels of gut hormones and gut barrier markers in male and female mice after eight-week feeding.

mRNA Level	SAW65	HAW35	HAW50	HAW65
**Male**				
**Cecum**				
Gut hormones				
Peptide YY	0.07 ± 0.01 ^b^	0.04 ± 0.01 ^a^	0.05 ± 0.01 ^ab^	0.06 ± 0.01 ^ab^
Proglucagon	0.03 ± 0.01	0.02 ± 0.01	0.03 ± 0.01	0.03 ± 0.01
Gut barrier markers				
Occludin	0.46 ± 0.03	0.40 ± 0.03	0.44 ± 0.02	0.41 ± 0.03
Mucin-2	3.34 ± 0.57	3.72 ± 0.70	3.19 ± 0.34	3.29 ± 0.87
**Colon**				
Gut hormones				
Peptide YY	0.21 ± 0.07	0.28 ± 0.07	0.23 ± 0.07	0.19 ± 0.02
Proglucagon	0.10 ± 0.02	0.11 ± 0.01	0.09 ± 0.02	0.11 ± 0.01
Gut barrier markers				
Occludin	0.60 ± 0.03	0.66 ± 0.05	0.69 ± 0.04	0.66 ± 0.03
Mucin-2	20.20 ± 3.96	19.03 ± 4.55	15.89 ± 3.75	22.43 ± 6.21
**Female**				
**Cecum**				
Gut hormones				
Peptide YY	0.05 ± 0.01	0.04 ± 0.01	0.06 ± 0.01	0.04 ± 0.01
Proglucagon	0.02 ± 0.01	0.01 ± 0.01	0.02 ± 0.01	0.02 ± 0.01
Gut barrier markers				
Occludin	0.31 ± 0.03	0.31 ± 0.04	0.31 ± 0.04	0.28 ± 0.03
Mucin-2	3.13 ± 0.35	2.40 ± 0.20	2.43 ± 0.29	2.12 ± 0.27
**Colon**				
Gut hormones				
Peptide YY	0.39 ± 0.06 ^ab^	0.39 ± 0.05 ^ab^	0.32 ± 0.06 ^a^	0.63 ± 0.09 ^b^
Proglucagon	0.14 ± 0.01	0.20 ± 0.03	0.16 ± 0.02	0.13 ± 0.01
Gut barrier markers				
Occludin	0.74 ± 0.04 ^ab^	0.67 ± 0.04 ^a^	0.74 ± 0.07 ^ab^	0.91 ± 0.05 ^b^
Mucin-2	18.68 ± 4.26	24.83 ± 3.81	25.54 ± 6.16	20.82 ± 8.34

Mice fed diets containing 65% standard amylose wheat (SAW65), 30% SAW and 35% high amylose wheat (HAW35), 15% SAW and 50% HAW (HAW50) or 65% HAW (HAW65). Data are shown as means ± SEM (*n* = 8 mice/group/sex). Values with different superscripts in a row indicate significant differences between groups (*p* < 0.05) as determined by one-way ANOVA and Tukey’s post-hoc test.

**Table 4:** Phylum-level relative abundances of fecal microbiota in male and female mice after eight-week feeding.

Phylum	SAW65	HAW35	HAW50	HAW65
Bacteroidetes	0.223 ^a^ (0.189, 0.262)	0.380 ^b^ (0.320, 0.425)	0.347 ^b^ (0.288, 0.381)	0.361 ^b^ (0.313, 0.425)
Firmicutes	0.713 ^b^ (0.680, 0.717)	0.552 ^a^ (0.499, 0.602)	0.571 ^a^ (0.504, 0.620)	0.556 ^a^ (0.508, 0.582)
Proteobacteria	0.0 (0.0, 0.0)	0.0 (0.0, 0.0)	0.0 (0.0, 0.0006)	0.0 (0.0, 0.0)
Actinobacteria	0.037 (0.028, 0.053)	0.038 (0.017, 0.057)	0.039 (0.030, 0.052)	0.040 (0.035, 0.054)
Patescibacteria	0.0 (0.0, 0.006)	0.0007 (0.0, 0.002)	0.001 (0.0, 0.003)	0.002 (0.0002, 0.002)
Tenericutes	0.001 (0.0, 0.007)	0.003 (0.0009, 0.005)	0.003 (0.0008, 0.009)	0.005 (0.002, 0.010)
Verrucomicrobia	0.005 (0.0, 0.012)	0.006 (0.0, 0.030)	0.025 (0.0, 0.034)	0.013 (0.0, 0.054)
Firmicutes: Bacteroidetes ratio	3.222 ^b^ (2.636, 3.779)	1.498 ^a^ (1.140, 1.873)	1.638 ^a^ (1.368, 2.111)	1.592 ^a^ (1.221, 1.887)
**Male**				
Bacteroidetes	0.224 ^a^ (0.189, 0.280)	0.352 ^b^ (0.319, 0.413)	0.369 ^b^ (0.336, 0.381)	0.363 ^b^ (0.346, 0.425)
Firmicutes	0.687 ^b^ (0.647, 0.723)	0.561 ^a^ (0.484, 0.613)	0.538 ^a^ (0.504, 0.574)	0.562 ^a^ (0.517, 0.589)
Proteobacteria	0.0 (0.0, 0.0)	0.0 (0.0, 0.0)	0.0 (0.0, 0.0009)	0.0 (0.0, 0.0)
Firmicutes: Bacteroidetes ratio	3.131 ^b^ (2.329, 3.779)	1.524 ^a^ (1.211, 1.918)	1.458 ^a^ (1.368, 1.694	1.541 ^a^ (1.221, 1.701)
**Female**				
Bacteroidetes	0.226 ^a^ (0.187, 0.249)	0.409 ^b^ (0.333, 0.446)	0.289 ^b^ (0.261, 0.387)	0.330 ^b^ (0.282, 0.482)
Firmicutes	0.719 ^b^ (0.688, 0.740)	0.547 ^a^ (0.499, 0.602)	0.619 ^a^ (0.475, 0.670)	0.553 ^a^ (0.427, 0.578)
Proteobacteria	0.0 (0.0, 0.0)	0.0 (0.0, 0.0005)	0.0 (0.0, 0.0004)	0.0 (0.0, 0.0)
Firmicutes: Bacteroidetes ratio	3.222 ^b^ (2.769, 3.984)	1.326 ^a^ (1.140, 1.821)	2.099 ^b^ (1.269, 2.570)	1.712 ^a^ (0.8803, 2.034)

Mice fed diets containing 65% standard amylose wheat (SAW65), 30% SAW and 35% high amylose wheat (HAW35), 15% SAW and 50% HAW (HAW50) or 65% HAW (HAW65). Data are shown as median ± IQR (*n* = 16 mice/group or 8 mice/group/sex). Values with different superscripts indicate significant differences between groups (*p* < 0.05) as determined by Kruskal-Wallis and pairwise comparisons tests with false discovery rate correction for *p*-values.

## Data Availability

The data presented in this study are available on request from the corresponding author.
